# Supplementary material for: Content-rich biological network constructed by mining PubMed abstracts
Source: BMC Bioinformatics. 2004 Oct 8;5:147. doi: 10.1186/1471-2105-5-147 (PMC528731; doi:10.1186/1471-2105-5-147)
Supplement: Additional File 2 — The original results of the above study (non-essential files are deleted to keep the file size under the limit set by BMC bioinformatics). [file 1471-2105-5-147-S2.bz2 › chilibotAdditionalFile2/dip10/352ID7834747E4685/html/BCL2_BAG1.html]

 


 **BCL2** and **BAG1** 
  
Found 100 abstracts in PubMed, retrieved 10.  
 

 What does Google say? 
 PDF only 
| .edu only 

---

**Interactive relationship** (e.g. stimulation, inhibition, etc)

**Neutral relationship**- Bag 1  [ **BAG1** ]  is a recently identified bcl 2  [ **BCL2** ]  related protein and is known to be linked to the biological aggressiveness of some carcinomas.  Ref: 12680148 Anticancer Res< MedlineTA>Anticancer Res,
- It was revealed that LIGHT treatment resulted in down regulation of anti apoptosis Bcl 2  [ **BCL2** ]  family member Bcl 2  [ **BCL2** ] , Bcl X L, Bag 1  [ **BAG1** ] , and Mcl 1.  Ref: 12767529 Cancer Lett, 2003
- gossypol induced apoptosis triggers down regulation of anti apoptosis Bcl 2  [ **BCL2** ]  members Bcl X L, Bag 1  [ **BAG1** ]  and Mcl 1, up regulation of pro apoptosis Bcl 2  [ **BCL2** ]  member Bak, activation of caspase,, and 9, up regulation of Apaf 1, release of cytochrome c cyto c from mitochondria, and activation of both DFF45 and PARP.  Ref: 12818369 Biochem Pharmacol, 2003
- CONCLUSION These findings suggest that bag 1  [ **BAG1** ]  may interact with bcl 2  [ **BCL2** ]  to play an important role in thyroid neoplasm before anaplastic transformation, and the disruption of events mediated by bag 1  [ **BAG1** ]  and bcl 2  [ **BCL2** ]  may be a typical characteristic of undifferentiated carcinoma.  Ref: 12680148 Anticancer Res< MedlineTA>Anticancer Res, 2003

**Non-interactive relationship** (e.g. studied together, co-existance, homology, etc.)

- MATERIALS AND METHODS We immunohistochemically investigated bag 1  [ **BAG1** ]  and bcl 2  [ **BCL2** ]  expression in various thyroid neoplasms using monoclonal antibodies.  Ref: 12680148 Anticancer Res< MedlineTA>Anticancer Res, 2003
- Bag 1  [ **BAG1** ]  expression in thyroid neoplasm its correlation with Bcl 2  [ **BCL2** ]  expression and carcinoma dedifferentiation.  Ref: 12680148 Anticancer Res< MedlineTA>Anticancer Res, 2003
- To determine whether changes in the expression of proteinases, 73 kDa constitutive heat shock cognate protein Hsc70 and stress inducible 72 kDa heat shock protein Hsp70 Hsc Hsp70, and Bcl 2  [ **BCL2** ]  associated gene product 1 BAG 1  [ **BAG1** ]  contribute to the remodeling response of muscle tissue, tibialis anterior muscles of male Sprague Dawley rats were injected with 0.75% bupivacaine and removed at, 1, or 35 days postinjection n = 5 7 group.  Ref: 12791605 Am J Physiol Endocrinol Metab, 2003
- The present study was performed to clarify the clinical implications of BAG 1  [ **BAG1** ] , a bcl 2  [ **BCL2** ]  binding protein in esophageal squamous cell carcinoma ESCC.  Ref: 12823208 Dis Esophagus, 2003
- The hsp70 hsc70 associating protein Hap46 of human origin, also called BAG  [ **BAG1** ]  1M Bcl 2  [ **BCL2** ]  associated athanogene 1, has been characterized previously as a DNA binding protein, which is able to stimulate transcription.  Ref: 12682371 Nucleic Acids Res, 2003
- Four anti apoptotic proteins were altered 2.1 to 14.6 fold for Bcl 2  [ **BCL2** ]  and BAG 1  [ **BAG1** ]  isoform p33 and p29.  Ref: 12706450 Eur J Pharmacol, 2003
- ... other Bcl 2  [ **BCL2** ]  family proteins including Bax, Bcl 2  [ **BCL2** ] , Bcl XL, and Bag  [ **BAG1** ]  remained unchanged.  Ref: 12874837 J Cell Biochem, 2003
